# The effect of a mindfulness-based intervention on executive, behavioural and socio-emotional competencies in very preterm young adolescents

**DOI:** 10.1038/s41598-021-98608-2

**Published:** 2021-10-06

**Authors:** Vanessa Siffredi, Maria Chiara Liverani, Petra Susan Hüppi, Lorena G. A. Freitas, Jiske De Albuquerque, Fanny Gimbert, Arnaud Merglen, Djalel Eddine Meskaldji, Cristina Borradori Tolsa, Russia Hà-Vinh Leuchter

**Affiliations:** 1 Division of Development and Growth, Department of Paediatrics, Gynaecology and Obstetrics, Geneva University Hospitals and University of Geneva, Geneva, Switzerland; 2 Institute of Bioengineering, Center for Neuroprosthetics, Ecole Polytechnique Fédérale de Lausanne, Lausanne, Switzerland; 3 Department of Radiology and Medical Informatics, Faculty of Medicine, University of Geneva, Geneva, Switzerland; 4 SensoriMotor, Affective and Social Development Laboratory, Faculty of Psychology and Educational Sciences, University of Geneva, Geneva, Switzerland; 5 CNRS, LPNC, University of Grenoble Alpes, 38000 Grenoble, France; 6 Division of General Paediatrics, Department of Paediatrics, Gynaecology and Obstetrics, Geneva University Hospitals and University of Geneva, Geneva, Switzerland; 7 Institute of Mathematics, Ecole Polytechnique Fédérale de Lausanne, Lausanne, Switzerland

**Keywords:** Preterm birth, Human behaviour, Paediatric research

## Abstract

Very preterm (VPT) children and adolescents show executive, behavioural and socio-emotional difficulties that persists into adulthood. This study aims to assess the effectiveness of a mindfulness-based intervention (MBI) in improving these competencies in VPT young adolescents using a randomised controlled trial design. 56 young adolescents aged 10–14 years, born before 32 gestational weeks, were randomly assigned to an “intervention” or a “waiting” group and completed an 8-week MBI in a cross-over design. Executive, behavioural and socio-emotional competencies were assessed at three different time points via parent and self-reported questionnaires, neuropsychological testing and computerised tasks. The data were analysed using an intention-to-treat approach with linear regression modelling. Our findings show a beneficial effect of MBI on executive, behavioural and socio-emotional competencies in VPT young adolescents measured by parent questionnaires. Increased executive competencies were also observed on computerised task with enhanced speed of processing after MBI. Two subgroups of participants were created based on measures of prematurity, which revealed increased long-term benefits in the moderate-risk that were not observed in the high-risk subgroups of VPT young adolescents. MBI seems a valuable tool for reducing detrimental consequences of prematurity in young adolescents, especially regarding executive, behavioural and socio-emotional difficulties.

*Clinical Trial Registration* ClinicalTrials, NCT04638101. Registered 20 November 2020—Retrospectively registered, https://clinicaltrials.gov/show/NCT04638101.

## Introduction

Follow-up studies indicate that very preterm (VPT) individuals are at increased risk for executive, behavioural and socio-emotional difficulties in childhood that persists into adolescence and adulthood^1–13^. Executive functioning (EF) is essential for goal-directed and adaptive problem-solving and behaviour. According to the model of Anderson^14^, it is conceptualised in four distinct subdomains: (1) attentional control, (2) information processing, (3) cognitive flexibility, and (4) goal setting. On the other hand, behavioural and socio-emotional competencies refer to a set of skills related to how individuals identify, express, understand, use and regulate their behaviour as well as their emotions and those of others^15^. Importantly, these competencies are crucial in daily life activities, with a close link to academic abilities and significant implications on social behaviour^16–19^.

These findings suggest that VPT children and adolescents may benefit from interventions designed to enhance executive, behavioural and socio-emotional competencies. In recent years, general interest in the practice and benefits of mindfulness-based interventions (MBI) has increased. Mindfulness is commonly defined as the on-going monitoring of present-moment experience while attending to it with openness, nonjudgment and acceptance^20^. In typically children and adolescents, mindfulness-based interventions have been associated with enhancement of all of executive subdomains, including attentional control, information processing, cognitive flexibility, and goal setting^21–30^. Similarly, MBI have been associated with improved emotional abilities, increased emotion regulation via reduction in stress, anxiety and social and behavioural problems, as well as greater empathy^24,31–38^. MBI have also been studied in clinical paediatric populations. In children from 8-year-old and adolescents with attention deficit hyperactivity disorder (ADHD), studies reported overall a beneficial effect of mindfulness-based or mindfulness-like interventions on ADHD symptoms^23,27,28,39–45^. These results were found both on objective measures of attention and executive functions as well as on self-reported, and parent/teacher-reported measures. It also appears that such interventions have effects lasting up to 8 weeks after the end of the MBI^42^. Finally, beneficial effects of MBI have been found in children from 7-year-old to adolescence suffering from social-emotional disorders, including anxiety, depressive and conduct disorders. These effects have been observed using a variety of self-reported and parent/teacher-reported measures, including measures of stress, anxiety, sleep and mental health^36,46–48^. Altogether, these studies suggest that MBI can be a valid way to support the development of executive functions, including attentional control and information processing speed, as well as behavioural and socio-emotional competencies. Mechanisms that underlie the beneficial effects of MBI remain unclear but evidence from recent adult behavioural and neuroimaging studies suggest that MBI relies on a process of enhanced overall self-regulation, including attention control, emotion regulation and self-awareness^49^.

This randomised controlled trial (RCT) study aims to assess the effectiveness of an 8-week MBI in VPT young adolescents aged 10–14 years to improve executive, behavioural and socio-emotional functioning. The age of 10–14 years has been targeted as a crucial developmental period during which MBI may be beneficial^50^.

## Results

### Neonatal and demographic characteristics

Neonatal and demographic characteristics of the 56 participants enrolled in the RCT are shown in Table 1. There were no significant differences in demographic and clinical characteristics at the age of 10–14 years between IG and WG (sex, age, index of general cognitive ability and socio-economic status) and the neonatal characteristics between IG and WG (gestational age, head circumference, length of hospitalisation, presence of severe brain lesions and other medical conditions).

**Table 1:** Neonatal and demographic characteristics at baseline of young adolescents enrolled in the RCT (n = 56), as well intervention group (IG) and waiting group (WG) comparisons.

	RCT, n = 56		
	Intervention group (IG), n = 29	Waiting group (WG), n = 27	Group comparison (IG vs. WG)
**Neonatal characteristics**			
Birth weight, mean (SD) [range] in grams	1284.83 (351.41) [650;1810]	1210 (400.85) [520;1980]	t(54) = 0.744, *p* = 0.460
Gestational age, mean (SD) [range] in days	29.29 (1.92) [24.71; 31.86]	29.12 (1.93) [26;31.71]	t(54) = 0.317, *p* = 0.753
Head circumference, mean (SD) [range] in cm	26.55 (2.57) [21;31]	25.65 (2.82) [21;31]	t(53) = 1.234, *p* = 0.223
Length of hospitalisation, mean (SD) [range] in days	59.56 (26.79) [23;131]	63 (33.69) [17;151]	t(52) = − 0.416, *p* = 0.679
Multiple births, n (%)	13 (44.8%)	7 (25.9%)	χ^2^(2) = 2.202, *p* = 0.333
cPVL, n(%)	1 (3.4%)	0	χ^2^(1) = 0.903, *p* = 0.342
IVH—Grades III and IV, n (%)	0 (0%)	0 (0%)	–
BPD, n (%)	5 (17.2%)	6 (22.2%)	χ^2^(1) = 0.534, *p* = 0.465
**Demographic characteristics**			
Sex			
Female, n	14 (48.3%)	16 (59.3%)	χ^2^(1) = 0.678, *p* = 0.410
Male, n	15 (51.7%)	11 (40.7%)	
Age at baseline, mean (SD) [range] in years	12.05 (1.23) [10.08;14.24]	12.26 (1.37) [10.38;14.85]	t(50) = − 0.585, *p* = 0.561
Index of general ability (GAI), mean (SD) [range]	106.67 (11.47) [83;132]	108.76 (11.23) [87;130]	t(50) = − 0.664, *p* = .0.510
Socio-economic status (SES), mean (SD) [range]	4.78 (2.62) [2;12]	3.76 (2.35) [2;12]	t(50) = 1.470, *p* = 0.148

Sex refers here to the individual's physical characteristics at birth associated with male or female. Independent-sample t-test, Chi-square was used to compare the randomised group.

*cPVL* cystic periventricular leukomalacia, *IVH* Intraventricular haemorrhage, *BPD* Bronchopulmonary dysplasia.

### RCT timing

Time differences (in days) between Time 1 and Time 2, as well as between Time 2 and Time 3 were not significantly different between the IG and the WG (*p* = 0.496, *p* = 0.502), Supplementary Table S2.

### Mindful attributes

There was no significant difference between before and after intervention for self-reported mindfulness attributes assessed by the Mindful Attention Awareness Scale Adapted for Children (MAAS-C; t(46) = 1.985, *p* = 0.053).

### Main outcomes

#### Executive competencies outcomes

**Figure 1:**
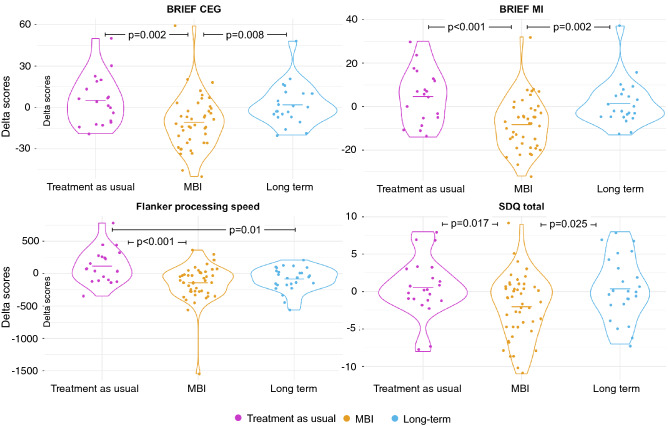
Plots showing the distribution of the delta scores (Δ) of the “Treatment as usual”, “MBI” and “Long-term” groups of the significant planned contrasts only. Lines in the violin plots represent the means for each group.

Planned contrasts “MBI” versus “treatment as usual” showed a significant effect of the MBI on the Behaviour Rating Inventory of Executive Function—parent version (BRIEF)^51^ for the Global Executive Composite (GEC) and Metacognition Index (MI) delta scores, reflecting enhanced executive capacities in everyday life (*p* = 0.002 and *p* < 0.001 respectively). This beneficial effect on executive functioning was supported by a significant decrease in delta reaction time on the processing speed measure of Flanker task (*p* < 0.001). Planned contrasts “MBI” versus “long-term” showed a significant increase for both BRIEF GEC and MI delta scores (*p* = 0.008 and *p* = 0.002), showing that the beneficial effect of MBI was not maintained three months after the end of the intervention. The planned contrast “treatment as usual” versus “long-term” showed a significant decrease in reaction time on the Flanker task processing speed measure (*p* = 0.01), reflecting a long-lasting effect of the MBI on this information processing subdomain, Fig. 1. There was no robust effect on other executive scores, including scores evaluated by parent-reported questionnaires, i.e., the Behavioural Regulation Index (BRI) from the BRIEF; and scores evaluated by neuropsychological testing, i.e., the letter-number sequencing task assessing working memory, the flanker inhibition score, the temporal context confusion index (TCC) assessing reality filtering and the Tempo Test Rekenen assessing timed mathematical achievement, Supplementary Tables S3.

#### Behavioural and socio-emotional competencies measures

The planned contrast “treatment as usual” versus “MBI” showed a significant effect of the MBI on the Strength and Difficulties Questionnaire—parent version (SDQ)^52,53^ for the delta total score with a significant decrease in scores after MBI (*p* = 0.017), reflecting an improvement in general behavioural competencies, Fig. 1. The planned contrast “MBI” versus “long-term” showed a significant increase in SDQ delta total score, showing that the beneficial effect of MBI was not maintained three months after the end of the intervention. There was no robust effect on other socio-emotional scores, including scores evaluated by self-reported questionnaires, i.e., total score of the KIDSCREEN-27 assessing quality of life, total score of the Social Goal Scale assessing social responsiveness and total score of the Self-Compassion Scale assessing self-compassion; and scores evaluated by neuropsychological testing, i.e., total score of the Affect Recognition subtest (NEPSY-II) assessing facial emotional recognition and the total score of the Theory of Mind subtest (NEPSY-II) measuring the ability to understand mental functions; Supplementary Tables S4.

### Subgrouping “prematurity”

Using K-means clustering, two groups of VPT participants were extracted based on weight and gestational age at birth: the high-risk group [n = 29, gestational age: mean (SD) = 27.91 (1.62); birth weight: mean (SD) = 938.1 (197.08)] and the moderate-risk group [n = 27, gestational age: mean (SD) = 30.63 (0.91); birth weight: mean (SD) = 1583.89 (196.8)].

#### Executive competencies outcomes

Planned contrasts “treatment as usual” versus “MBI” showed a significant effect of the MBI in both the high- and moderate-risk subgroups for the BRIEF MI (high-risk, *p* = 0.016; moderate-risk, *p* = 0.003) with a significant decrease of BRIEF MI delta scores; as well as a decrease in BRIEF GEC deltas scores only in the high-risk subgroup (*p* = 0.011). The planned contrasts “MBI” versus “long-term” and “treatment as usual” versus “long-term” showed a significant increase in the BRIEF MI and CEG delta scores three months after MBI in the high-risk subgroup only, reflecting that the beneficial effect of MBI was not maintained in this group, Fig. 1. For both subgroups, planned contrasts “treatment as usual” versus “MBI” showed a significant decrease in delta reaction time on the Flanker task, reflecting increased processing speed after MBI (high-risk, *p* = 0.035; moderate-risk, *p* = 0.001). In the moderate-risk subgroup only, planned contrasts “treatment as usual” versus “long-term” showed a significant decrease in reaction time on the Flanker task, reflecting an increase in processing speed that lasted 3 months after the end of the MBI (*p* = 0.001), Fig. 1. There was no robust effect for the other executive scores, including scores evaluated by parent-reported questionnaires, i.e., the Behavioural Regulation Index (BRI) from the BRIEF; and scores evaluated by neuropsychological testing, i.e., the letter-number sequencing task assessing working memory, the flanker inhibition score, the temporal context confusion index (TCC) assessing reality filtering and the Tempo Test Rekenen assessing timed mathematical achievement, Supplementary Tables S5 and S6.

#### Behavioural and socio-emotional competencies outcomes

**Figure 2:**
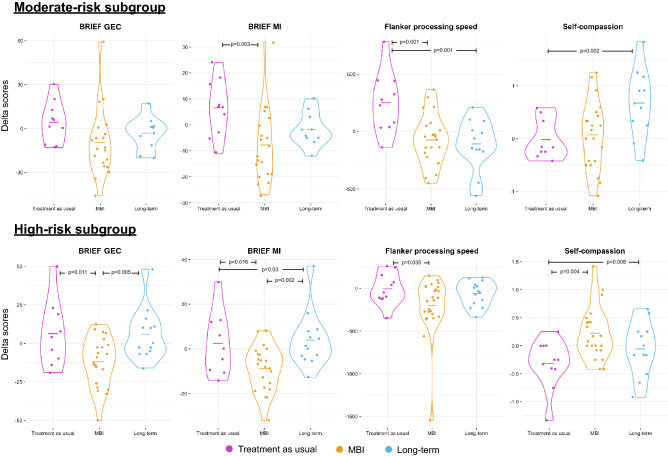
Distribution of the delta scores (Δ) of the “Treatment as usual”, “MBI” and “Long-term” groups for the significant planned contrasts for the two subgroups of VPT: moderate-risk and high-risk. Lines in the violin plots represent the means for each group.

For significant linear regression models adjusted for multiple comparisons, planned contrasts “treatment as usual” versus “MBI” showed a significant increase in self-compassion delta scores after MBI specific to the high-risk subgroup (*p* = 0.004), reflecting enhanced self-compassion after MBI, Fig. 2. For both the moderate- and the high-risk subgroups, planned contrasts “treatment as usual” versus “long-term” showed a significant increase in self-compassion scores 3 months after the end of the intervention (moderate-risk, *p* = 0.002; high-risk, *p* = 0.008). There was no robust effect on other socio-emotional scores, including scores evaluated by parent-reported questionnaires, i.e., total score of the SDQ assessing behavioural difficulties; scores evaluated by self-reported questionnaires, i.e., total score of the KIDSCREEN-27 assessing quality of life and total score of the Social Goal Scale assessing social responsiveness; and scores evaluated by neuropsychological testing, i.e., total score of the Affect Recognition subtest (NEPSY-II) assessing facial emotional recognition and the total score of the Theory of Mind subtest (NEPSY-II) measuring the ability to understand mental functions, Supplementary Tables S5 and S6.

## Discussion

This RCT assessed the effectiveness of an 8-week MBI in VPT young adolescents to improve executive, behavioural and socio-emotional competencies. Our findings show beneficial effects of MBI immediately after the intervention on executive, behavioural and socio-emotional competencies in every-day life based on parent-reported questionnaires and on processing speed capacities. Subgrouping analyses based on the level of prematurity reveal a larger beneficial effect of MBI immediately after the intervention in the high-risk VPT subgroup, but larger long-lasting effects of the MBI in the moderate-risk VPT subgroup. Our findings suggest that the use of MBI in VPT young adolescents is effective in improving executive as well as behavioural and socio-emotional outcomes.

Parent-reported questionnaires revealed an increase in overall executive competencies in everyday life, together with a more specific effect on metacognitive abilities. An enhancement of processing speed on a computerised task corroborates these results reflecting increased skills in the information processing EF subdomains^14^. These findings are in line with previous studies conducted in different populations of children and adolescents showing strong effect of MBI on processing speed^30,54–56^. Although we found a long-lasting beneficial effect of MBI 3 months’ post-intervention on processing speed capacities, the beneficial effect of MBI on overall executive and behavioural competencies reported by parents, was not maintained. Subgrouping analyses based on prematurity levels gave valuable insight into these results. In fact, regarding executive competencies, the high-risk subgroup appears to benefit slightly better from the MBI immediately post-intervention, with greater enhancement of overall executive competencies in daily life, in addition to improvements in metacognitive abilities and processing speed compared to the moderate-risk group. Nevertheless, the decline in executive competencies observed 3 months post-MBI seems mostly driven by the high-risk subgroup. At the opposite, the long-lasting effect of MBI on processing speed was found only in the moderate-risk group. Finally, other measures of executive competencies evaluated through neuropsychological testing did not show significant changes after MBI, including inhibition, working memory and reality filtering competencies. These findings are particularly surprising for inhibition and working memory as these execution functions have showed solid associations with MBI in previous children and adolescents studies^57^. For inhibitory competencies as measured by the Flanker Visual Filtering Task, our results are inconsistent with previous studies using this same task who found effect of MBI in selective attention and inhibition in children from 3 to 7-year-old and from 9 to 11-year-old^31,58^. A possible explanation for these inconsistent findings might be that the task in the present study was not cognitively demanding enough for our older population of young adolescents, as indicated by the presence of a ceiling effect on task’s performance. Further exploration using a Flanker task with an adapted level of difficulty, i.e., more demanding, should be done to clarify this point. In regards to working memory, previous studies found beneficial effect of MBI using parent questionnaires and evaluating working memory in daily life^59^. This is consistent with our results on parent’s questionnaire showing a more specific effect of MBI on the BRIEF metacognition score comprising a Working Memory subscore. Nevertheless, it is possible that neuropsychological tests used in the present study (e.g., letter-number sequencing task) was not sufficiently fine-grained to capture beneficial effects of the intervention on working memory.

When exploring behavioural and socio-emotional competencies, our results showed a significant improvement immediately after MBI only on the total score of the SDQ parent-reported questionnaire, reflecting an improvement in general behaviour. These findings are in line with previous research showing enhancement of behavioural competencies after MBI during adolescence^41,60,61^. Nevertheless, this effect was not maintained 3 months after the end of the intervention. In regards to self-compassion (self-reported questionnaire), the subgrouping analyses revealed a significant improvement immediately after the MBI only in the high-risk VPT group. In contrast, a significant improvement three months after the end of the MBI was observed in both the high- and moderate-risk groups. Moreover, we did not observe any significant effect of the MBI on quality of life and social responsiveness as assessed by self-reported questionnaires, nor on affect recognition and theory of mind using neuropsychological testing. In previous studies, MBI beneficial effects on quality of life and social responsiveness were evaluated by parents^62^. It is possible that when evaluated by the adolescents themselves, the relationship between quality of life and social responsiveness with MBI is mediated by other factors, such as family functioning^63^. In regards to the absence of an effect of MBI on affect recognition and theory of mind, this is partially consistent with previous studies^64^. However, it is possible that the standardised tests used in the context of the study might not be sensitive enough to detect small changes in socio-emotional competencies.

Our study has several strengths. We used a gold standard RCT design, recruited a relatively large sample of VPT young adolescents and analysed the data on an intention-to-treat basis. Nevertheless, theoretical and methodological limitations of this study should inform future research. First, the absence of an active control condition or a placebo condition is an important limitation to this study. It has been suggested that when control groups do not experience any new and exciting activity, in case for example of a wait-group control comparison, improvements that occur for the treatment group cannot be attributed to mechanisms beyond nonspecific effects of novelty^65,66^. Therefore, comparing MBI to an equally engaging active control condition is necessary to provide reliable results and a better understanding of what factors may contribute to the beneficial effect of MBI^67^. Secondly, the beneficial effect of MBI observed via parent-reported questionnaires is questionable. The subjective aspect of these tools is well-documented and parents were not blind to the intervention^68^. In this context, the use of an active control would also allow to control for any placebo effect that the MBI could have induced. This would allow participants and their families to be blinded to treatment allocation, as well as help understand what effects are specifically attributable to MBI. Moreover, future studies should consider the completion of questionnaires by multiple informants from different settings (e.g. parents and teachers) to give a more objective view of the changes occurring after MBI^41,69^. Thirdly, in our study, there was no change before and after MBI on mindfulness attributes as measured by the MAAS-C. This instrument, as most instruments measuring mindfulness attributes, probably captures only some variations, components or aspects of mindfulness and leaves others out^70^. Previous research also suggested that this scale may be inadequate to represent intentional attention or awareness^71^. Therefore, it is possible that the absence of difference before and after intervention on the MAAS-C might not fully reflect the evolution of the participants’ mindfulness attributes. Finally, factors such as home environment, caregiver involvement, and motivation to participate in the training and quantity of home practice were not considered in our study^72,73^. This might influence the outcomes of an MBI and should be considered in future research.

In conclusion, this study shows for the first-time beneficial effects of MBI in VPT young adolescents on enhancing executive, behavioural and socio-emotional competencies. Subgrouping analyses based on prematurity level reveal a larger beneficial effect of MBI immediately post-intervention in the high-risk subgroup, but a larger long-lasting effect of MBI in the moderate-risk subgroup. We conclude that the use of MBI in VPT young adolescents is effective in improving executive, behavioural and socio-emotional outcomes. However, a longer MBI intervention might be beneficial for high-risk VPT young adolescents. Although future investigations are needed, MBI seems a promising tool to enhance executive, behavioural and socio-emotional outcomes in a vulnerable population such as VPT young adolescents.

## Methods

The “Mindful preterm teens” study is an RCT of an MBI in VPT adolescents aged 10–14 years, see Siffredi, Liverani and colleagues for a detailed description^74^. All experimental protocols were approved by the Swiss Ethics Committees on research involving humans, ID: 2015-00175. All methods were carried out in accordance with relevant guidelines and regulations. Written informed consent was obtained from primary caregivers and participants.

### Participants

**Figure 3:**
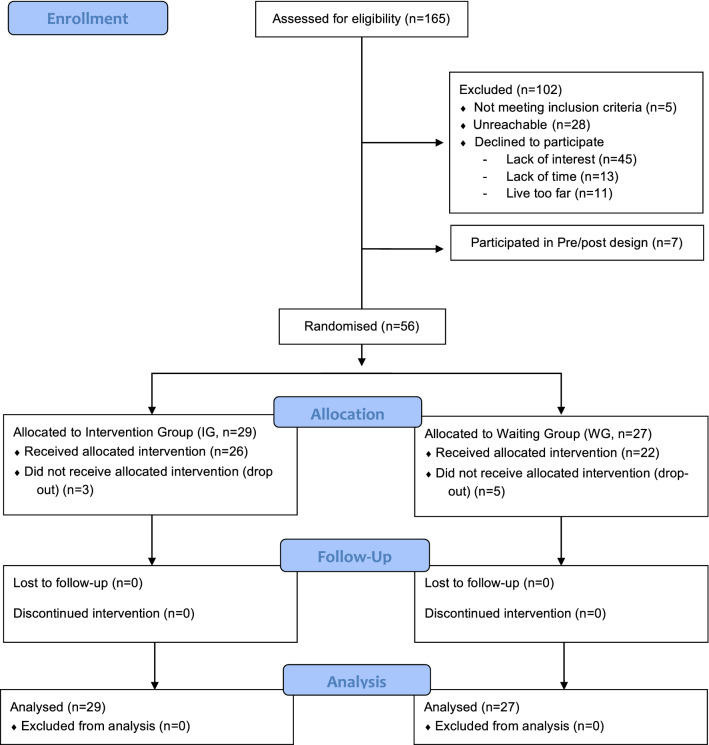
CONSORT flow diagram of the present cross-over RCT design.

One hundred and sixty-five VPT young adolescents were invited to participate in the study. They were aged 10–14 years, born before 32 gestational weeks between 01.01.2003 and 31.12.2008 in the Neonatal Unit at the Geneva University Hospital, Switzerland, and received follow-up care at the Division of Child Development and Growth at the Geneva University Hospital. VPT young adolescents were excluded if they had an intelligence quotient below 70, sensory or physical disabilities (cerebral palsy, blindness, hearing loss), or an insufficient understanding of French. Moreover, some families declined to participate due to lack of time, lack of interest, geographical constraints or unreachability. Out of the 165 young adolescents invited to participate, 56 (33.9%) were enrolled in the RCT, Fig. 3.

### Procedures

**Figure 4:**
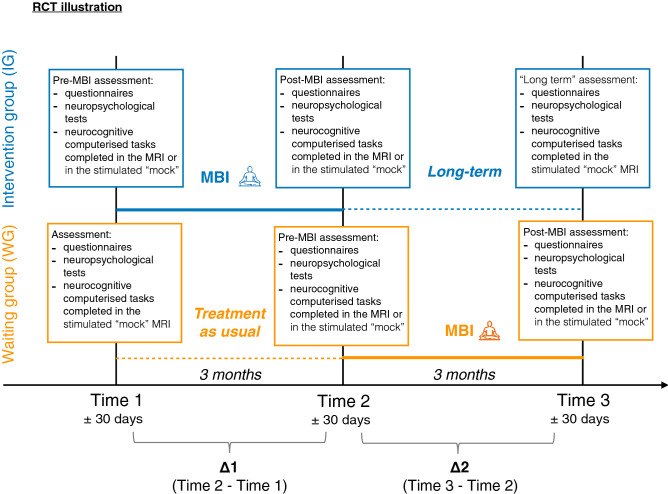
Illustration of the RCT study design. Participants enrolled in the RCT design were randomised in two groups: the intervention group (IG) in blue and the waiting group (WG) in orange.

Once enrolled in the RCT, families were allocated to the intervention group (IG) or the waiting group (WG) with a cross-over RCT design, Fig. 4. An independent biostatistician generated a random number table. Families were allocated to the next available sequential study number which corresponded to an opaque sealed envelope which contained the randomisation allocation to the IG or WG. The project coordinators or research assistants opened the envelope to obtain the group allocation after enrolment and before the first appointment. To facilitate the participation of families with twins, the randomisation was completed for a single family, so that siblings would be consequently allocated in the same group.

All participants completed a baseline assessment to evaluate general intellectual functioning and demographic characteristics. Additional assessments were completed at three different time points, where outcome measures were collected via parent-reported and self-reported questionnaires, neuropsychological assessments and computerised neurocognitive tasks. Children from the IG completed the MBI between Time 1 and Time 2. Participants from the WG completed the MBI between Time 2 and Time 3. For all young adolescents involved in the trial, the pre-intervention assessment (i.e., Time 1 for the IG, and Time 2 for the WG) was completed within 1 month before the first MBI session. The post-intervention assessment (i.e., Time 2 for the IG, and Time 3 for the WG) was completed within 1 month after the last MBI session. For the IG, the remaining assessment (i.e., Time 3) was completed 3 months after the post-intervention assessment and will be referred to as “Long term” assessment. For the WG, the remaining assessment (i.e., Time 1) was completed three months before the pre-intervention assessment.

### Mindfulness-based intervention and mindful attributes

MBI consisted of eight weekly sessions in groups of up to seven participants, lasting 90 min, as well as an invitation to practice daily at home. Two instructors were present for each group throughout the intervention. The MBI program used in this study was specifically adapted to adolescents, Supplementary Methods^74^.

The Mindful Attention Awareness Scale Adapted for Children (MAAS-C) was used to assessed mindful attributes using a 6-point Likert-type scale ranging from (1) almost never to (6) almost always, where higher scores reflect higher mindfulness attributes^75^. The MAAS-C was completed before and after MBI by the participants who completed the MBI.

### Neonatal and demographic characteristics

Neonatal characteristics were documented from medical records. In order to estimate general intellectual functioning, the General Ability Index (GAI) from the Wechsler Intelligence Scale for Children-4th Edition (WISC-IV) was used^76^. Parent-reported and self-reported demographic questionnaires were used to assess general characteristics of the participants. Socio-economic status was estimated from maternal education and paternal occupation using the validated Largo scale. Higher socio-economic scores reflect lower socio-economic status levels^77^.

### Outcome measures

Participants’ executive, behavioural and socio-emotional functioning were assessed using parent-reported and self-reported questionnaires, neuropsychological testing and computerised neurocognitive tasks, Supplementary Table S1.

#### Executive competencies measures

Executive competencies of young adolescents were assessed using the Behaviour Rating Inventory of Executive Function—parent version (BRIEF) evaluating attention, hyperactivity and impulsivity in everyday life^51^. The BRIEF comprises 86 items over two standardised subscales, the Behavioural Regulation Index (BRI) and the Metacognition Index (MI), as well as a global score called the Global Executive Composite (GEC). Neurocognitive computerised tasks comprised: (1) the Flanker Visual Filtering Task, in which reaction time of the congruent condition was used to assess speed of processing, which belongs to the information processing subdomain, and the inhibition score (reaction time in incongruent conditions–reaction time in congruent conditions) was used as a measure of the attentional control subdomain^14,78^; (2) the child-adapted version of the Reality Filtering task, in which the temporal context confusion index (TCC) was used as a reality filtering measure, which involves integration of different executive processes^79,80^. Neuropsychological testing included the Letter-Number Sequencing subtest from WISC-IV assessing working memory, which belongs to the cognitive flexibility subdomain^14^. Given the strong association between executive functions and mathematical abilities in children and adolescents^81,82^, we also used the total score of the Tempo Test Rekenen to assess timed mathematical achievement^83^.

#### Behavioural and socio-emotional competencies measures

The total score of the Strength and Difficulties Questionnaire—parent version (SDQ) was used to assess behaviour in daily life^52,53^. Participants completed three self-reported questionnaires: the KIDSCREEN-27 items questionnaire was used to assess the quality of life of the participants^84^; the total score of the Social Goal Scale was used to assess social responsiveness and social relationships^85^ and the total score of the Self-Compassion Scale—Short form was used to assess the main components of self-compassion^86^.

Neuropsychological testing included the Affect Recognition subtest (NEPSY-II) giving a total score assessing facial emotional recognition; and the Theory of Mind subtest (NEPSY-II) giving a total score measuring the ability to understand mental functions, such as belief, intention or deception^87^.

### Statistical analyses

#### Main statistical analyses

To explore differences in the MAAS-C questionnaire before and after the intervention, paired-sample t-tests were used.

All analyses on outcome measures were based on the intention-to-treat principle. For each outcome measure, raw scores were used to calculate differences between Time 1 and Time 2 (Time 2-Time 1 = Δ1), and between Time 2 and Time 3 (Time 3 − Time 2 = Δ2) for each participant, Fig. 4. Negative Δ indicates a reduction of the scores between two time points, whereas positive Δ indicates an increase in scores between two time points. Linear regression models were used to evaluate the effect of MBI. Assumptions of linear regression models were assessed based on visual diagnosis of the distribution of the residuals. We modelled fixed effects of outcome measures as dependent variables and interaction of time (i.e., Δ1 and Δ2) by group (i.e., IG and WG) as independent variables. When the model’s *p* value was significant, we used planned contrasts to compare outcome measures between the different levels of the independent variables time and group:

- we assessed the effect of the intervention immediately after MBI using the planned contrast defined as: “MBI” (i.e., Δ1 of IG and Δ2 of WG) versus “treatment as usual” (i.e., Δ1 of WG).
- we assessed delayed effect of MBI using the planned contrast defined as: “long-term” (i.e., Δ2 of IG) versus “treatment as usual” (i.e., Δ1 of WG).
- when the effect of the intervention immediately after MBI was significant (“MBI” vs, “treatment as usual”), we assessed the long-term effect of the intervention using the planned contrast defined as: “MBI” (i.e., Δ1 of IG and Δ2 of WG) versus “long-term” (i.e., Δ2 of IG).

Effect size and *p* values were calculated. The *p* values were also corrected for multiple comparisons using the Benjamini and Hochberg method (1995), which controls the False Discovery Rate correction (FDR, q values ≤ 0.05)^88^. All analyses were performed using R software, version 3.5.2^89,90^. Of note, as this was not in the scope of this manuscript, associations between self-reported and neurocognitive testing were not explored.

#### Subgrouping “prematurity” analyses

In order to better understand inter-individual differences, we performed exploratory analyses on specific subgroups of VPT young adolescents. Clustering analyses were used to explore whether any treatment effect tested in our RCT varied across subgroups defined by pre-intervention patient characteristics^91^. Subgrouping of participants was determined by K-means clustering and was based on the main properties of premature birth. A subgrouping “prematurity” was created by using the measures of birth weight and gestational age as features to create two groups of VPT participants: the “high-risk” group, including participants with lower birth weight and lower gestational age, and the “moderate-risk” group, including participants with higher birth weight and higher gestational age. To evaluate the effect of MBI on these subgroups, analyses similar to the section above were conducted.

## Supplementary Information

Supplementary Information.

## Data Availability

Deidentified individual participant data (including data dictionaries) will be made available, in addition to study protocols, the statistical analysis plan, and the informed consent form. The data will be made available upon publication to researchers who provide a methodologically sound proposal for use in achieving the goals of the approved proposal. Proposals should be submitted to Russia.HaVinhLeuchter@unige.ch.
